# The Role of Colonic Bacteria in the Metabolism of the Natural Isoflavone Daidzin to Equol

**DOI:** 10.3390/metabo5010056

**Published:** 2015-01-14

**Authors:** Fatemeh Rafii

**Affiliations:** Division of Microbiology, National Center for Toxicological Research, FDA, Jefferson, AR 72079, USA; E-Mail: Fatemeh.Rafii@fda.hhs.gov; Tel.: +1-870-543-7342; Fax: +1-870-543-7307

**Keywords:** metabolites, metabolism, intestinal bacteria, phytoestrogen, isoflavone, daidzin, daidzein, equol

## Abstract

Isoflavones are found in leguminous plants, especially soybeans. They have a structural similarity to natural estrogens, which enables them to bind to estrogen receptors and elicit biological activities similar to natural estrogens. They have been suggested to be beneficial for the prevention and therapy of hormone-dependent diseases. After soy products are consumed, the bacteria of the intestinal microflora metabolize isoflavones to metabolites with altered absorption, bioavailability, and estrogenic characteristics. Variations in the effect of soy products have been correlated with the isoflavone metabolites found in plasma and urine samples of the individuals consuming soy products. The beneficial effects of the soy isoflavone daidzin, the glycoside of daidzein, have been reported in individuals producing equol, a reduction product of daidzein produced by specific colonic bacteria in individuals called equol producers. These individuals comprise 30% and 60% of populations consuming Western and soy-rich Asian diets, respectively. Since the higher percentage of equol producers in populations consuming soy-rich diets is correlated with a lower incidence of hormone-dependent diseases, considerable efforts have been made to detect the specific colonic bacteria involved in the metabolism of daidzein to the more estrogenic compound, equol, which should facilitate the investigation of the metabolic activities related to this compound.

## 1. Introduction

The community of microorganisms residing in the human gastrointestinal tract forms a complex ecosystem populated with over 10^11^–10^12^ bacterial cells per gram of contents of the large intestine [1,2,3]. The colon harbors more than 400 bacterial species, the majority of which are anaerobes; they are present both in the colonic contents and attached to the mucosa [4]. The diverse community of microorganisms in the colon has coevolved with the human host, with which it has a mutually beneficial relationship [1,3,5]. The colonic environment provides nutrients, pH, temperature, and redox potential favorable for the biological and biochemical reactions of the microbial community [4]. The microbial community acts as a barrier against pathogenic bacteria that might colonize the colon, and it metabolizes nutrients, drugs, and xenobiotics. Microbial metabolism by colonic bacteria is reported to contribute to the metabolic, nutritional, physiological, and immunological processes of the host; the extent of this contribution to human health is unknown [2].

The collective genome of the human intestinal microbiota is comprised of 3.3 million genes, approximately 150 times more genes than are in the human genome [2]. The metabolome, or the metabolic signature of an individual, is dependent not only upon the host but also upon the interaction of the host with the microflora [2,3,5,6]. Some of the metabolites found in a host are exclusively the result of the activities of bacteria in the colonic microbial community. This includes the digestion of fibers not metabolized by host enzymes into short-chain fatty acids, and the metabolism of isoflavones, which is performed by a number of bacteria [2,3,5]. The types of colonic bacteria metabolizing isoflavones in each individual affect the outcome of the consumption of isoflavones [2,3]. Inter-subject differences in response to isoflavones may be due to specific differences in gut microflora species [7,8,9,10,11,12,13,14,15,16]. In this mini-review, the role of colonic bacteria in the metabolism of the isoflavone daidzin will be discussed. 

## 2. Isoflavone Phytoestrogens

Isoflavone phytoestrogens are bioactive plant constituents that are found, mainly in conjugated forms, in the products of more than 40 plant species, including soybeans, other legumes, whole grains, berries, and nuts [12,17,18,19,20,21]. Two of the widely studied isoflavones from soybeans are daidzin and genistin, the glycoside forms of daidzein and genistein, respectively. They are found in different concentrations depending on the growth conditions [21]. Among other naturally occurring isoflavonoids from legumes are biochanin A, formononetin, and glycitein, the methoxylated forms of genistein, daidzein, and 6,7,4’-trihydroxyisoflavone, respectively [22].

Although isoflavones are nonsteroidal, they have similarities in chemical structure to mammalian estrogens, show estrogenic activities in biological assays, and induce estrogen-like effects in mammalian systems [23,24,25,26,27]. In legume plants, daidzin and genistin act as signal molecules for symbiotic nitrogen-fixing bacteria [28]. In mammalian systems, they have numerous biological activities and may also be metabolized, which alters their effects [29,30].

## 3. Role of Dietary Isoflavones

The soy-rich diets of Asian populations contain high levels of isoflavones, which are believed to contribute to the lower incidence of hormone-dependent diseases, cardiovascular diseases, breast, prostate and colon cancer, and menopausal symptoms, including osteoporosis [12,29,31,32,33,34,35,36]. The rate of breast cancer in Asian women is one third to one half of that in Caucasians [29] and the frequency of menopausal hot flashes in Asian women is less than that of Western women [11,32,33,34,37]. Adoption of Western diets by immigrant Asians has put them at higher risk of breast and prostate cancer, suggesting that dietary and environmental factors are more important than racial characteristics [38]. The beneficial effects of dietary isoflavones in soy are not always clear from *in vivo* studies [29], but health benefits have been reported in more than half of the clinical studies [16,39].

Variations observed in the effects of phytoestrogens depend on various factors, including differences in the population of bacteria in the intestinal tract capable of metabolizing isoflavones. Observation and interventional studies have shown the ability of the intestinal microfloras of some individuals to convert isoflavones to metabolites that may result in a reduced risk of hormone-dependent diseases [12,39,40,41,42,43,44,45,46,47].

## 4. Role of Intestinal Microflora in the Bioavailabilities of Isoflavones

### 4.1. Intestinal Bacteria

Bacteria in both the small and the large intestine are involved in the metabolism of natural isoflavones [18,22,48,49,50]. The microbial hydrolytic enzymes are involved in the deconjugation of natural isoflavones (primary metabolism) and release of more bioavailable products. The bacteria converting primary metabolites to more or less estrogenic secondary metabolites are mainly found in the colon [18,22,31,36,48,49,50].

### 4.2. Hydrolyis of Isoflavones

The intestinal microflora has a major role in the metabolism, bioavailability, biological activities, and metabolomic profiles of dietary isoflavones. Soybeans contain the natural isoflavone glycosides, daidzin and genistin, which because of their large hydrophilic structures are not easily absorbed across the enterocytes or intestinal absorptive cells [18,22,31,36,48,49,50,51,52]. However, intestinal bacteria produce hydrolytic enzymes capable of hydrolyzing the natural isoflavones and releasing the unconjugated isoflavones, daidzein and genistein, which are more estrogenic and readily absorbed [31,49,51,52,53,54,55]. In addition to the glucosidase produced from the intestinal brush border [56], β-glucosidases of bacteria in the small intestine have a high affinity for daidzin and genistin, hydrolyzing them to their aglycones. Deglycosylation of these compounds is an important step in the absorption, metabolism, excretion, and biological activities of isoflavones [54,56]. 

After daidzein and genistein enter the circulation via absorption through the intestine, they may be transported to the liver, where they are conjugated by the liver enzymes to form sulfated, glycosylated and glucuronidated isoflavones, which are more water-soluble [57]. Gu *et al.* [57] showed that isoflavones in human plasma are predominantly glucuronides (75%), sulfates (24%), and aglycones (1%).

### 4.3. Metabolism of Isoflavones by Colonic Bacteria

Metabolism of isoflavones by the colonic microflora alters their biological activities [31,54,58,59,60,61,62]. The liver conjugates of isoflavones may be excreted in the bile and pass into the intestinal lumen [56,63]. These isoflavones, along with ingested and deconjugated isoflavones in the small intestine, enter the colon, where they are subjected to further hydrolysis and microbial transformation by the colonic microflora [56,63]. 

**Figure 1:**
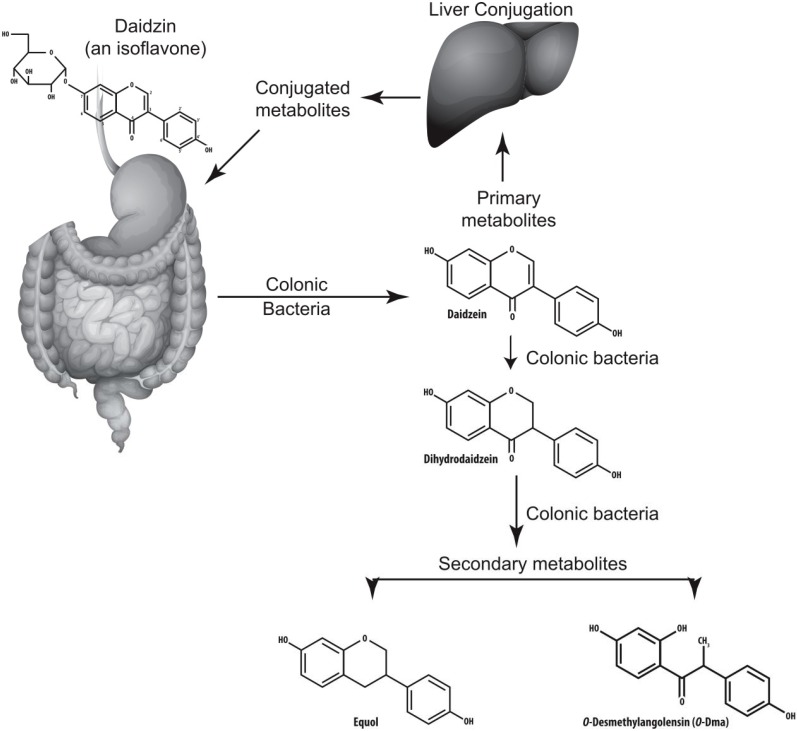
Illustration of the role of colonic bacteria in the production of primary and secondary metabolites from the isoflavone daidzin and in the metabolism and circulation of daidzein liver conjugates. Whereas the intestinal microflora produces (*S*)-equol from daidzein, the *O*-desmethylangolensin (DMA) produced as the result of intestinal bacterial activities is (*R*)-*O*-DMA.

Enzymes from the human colonic microflora deconjugate conjugated isoflavones, which are then reabsorbed through the enterohepatic circulation, altering their pharmacokinetic distribution, or metabolize them further to compounds that are either more or less estrogenic than the parent compounds (Figure 1) [29,64]. Thus, the host colonic bacteria are important in the bioavailabilities and biological activities of isoflavones and influence the metabolomic profile of the host after consumption of isoflavones [64,65]. Some of the intestinal bacterial strains that are involved in the metabolism of the isoflavone daidzin (Figure 1) have been identified and their roles in the production of primary and secondary metabolites are described in the following sections. 

One of the widely studied metabolites produced by bacteria residing in the gut of subjects consuming soy-containing isoflavones is equol, which until recently was thought to be produced only by anaerobic colonic bacteria [31,54]. Only 30% of the western population carries colonic bacteria that convert daidzein to equol. This population, which is designated as equol producers [7,13,15,64,65,66], may benefit from consumption of soy products more than non-producers, which may convert daidzein to a less estrogenic metabolite, *O*-desmethylangolensin (*O*-DMA) [29]. The serum equol concentration in nonproducers is ≤40 nmol/L, which is much lower than the ≥83 nmol/L found in the serum of equol producers [36]. The presence of 60% equol producers in Asian populations, *versus* the 30% in Western populations, is associated with a lower incidence of hormone-dependent diseases among this population that also consumes more dietary isoflavones through a soy-based diet [29,54,55]. In the following sections, equol, the biologically important metabolite of daidzein, and the intestinal bacteria involved in the metabolism of natural isoflavones and biosynthesis of equol are discussed.

## 5. Equol

One of the most widely studied metabolites of the isoflavone daidzein is equol, (3*S*)-3-(4-hydroxyphenyl)-7-chromanol, which is produced by bacteria that have specialized enzymes [7,14,16,29,54,55]. The structure has two phenolic rings, with reactive hydroxyl groups, and a central furan ring with an inert oxygen (Figure 1). It is insoluble in water and acid-labile. Synthetic equol exists as two enantiomers, (*S*)-(−)-equol and (*R*)-(+)-equol, both of which are biologically active and modulate androgen action, but only *S*-equol occurs naturally [54]. 

*S*-Equol is produced by the action of intestinal bacteria on daidzein in 20%–30% of the population in Western countries and 50%–60% of Asian populations consuming soy-containing diets [54,55]. Because of its enhanced ability to bind to estrogen receptors, equol is one of the most bioactive isoflavones [9,23,24,45,47,54,55,67,68,69,70,71,72]. After consumption of a diet containing soy, equol is found as a metabolic by-product of the activity of colonic intestinal bacteria in human equol producers [7,36,54,55,66]. 

Equol has an affinity for the human estrogen receptors ERα and ERβ, strong antioxidant activities, and other properties [9,36,69,70,73,74,75,76,77]. Equol is readily absorbed through the colonic wall, but the rate of its clearance from plasma is slower than that of daidzein [15]. The production of equol in humans is solely the result of the intestinal bacterial metabolism of daidzein [36,54,55]. Daidzein, *O*-DMA, and equol bind to and activate the estrogen receptors ERα and ERβ so they will translocate to the nucleus and bind to the estrogen response element, a specific DNA sequence with high affinity for estrogen receptors located in or near the promoter region of target genes [42,67]. The binding of activated ERα and ERβ to the estrogen response element is the first step in the induction of gene activation and transcription [42]. The affinity of *O*-DMA and equol for ERα and ERβ is higher than that of daidzein [78,79,80]. Equol is able to activate the binding of both ERα and ERβ to the estrogen response element better than daidzein, which preferentially activates binding of ERβ to the estrogen response element [81].

Markiewicz *et al.* [78] showed that equol was more estrogenic for human adenocarcinoma cells than daidzein. Both *O*-DMA and equol stimulate the growth of estrogen-responsive MCF-7 breast cancer cells more than daidzein [31]. It has been shown that equol has higher antioxidant activity than daidzein [9,36,69,73,75,76,77,82,83,84,85]. Studies have indicated that equol is better than daidzein for inhibiting the proliferation of benign and malignant prostatic cell lines [86]. Equol is 100 times stronger than daidzein in stimulating the mRNA expression of an estrogen-responsive protein (pS2) in MCF-7 cells [80]. Exposure of MCF-7 cells to both equol and 17β-estradiol reduces the expression of pS2 mRNA below the level of expression of this gene with estradiol alone, indicating that equol has antiestrogenic activity [80]. Equol, whether produced by the microbiota or directly administered to female mice, is also anti-estrogenic in the reproductive tissue [87]. Administration of equol and endogenously produced equol was shown to reduce the expression of the progesterone receptor in the mice vaginal epithelium, indicating that equol reduces the tissue estrogen-dependent response [87].

## 6. Colonic Bacteria Associated with the Metabolism of Isoflavones

Metabolites of phytoestrogens have been detected in the urine of humans and experimental animals [15]. They are also found as the products of colonic microflora in human flora-associated-germ-free rats [17,88]. The levels of metabolites of isoflavones (equol and *O*-DMA ) that are found in germ-free rats after association with the colonic microflora of humans are dependent on the amounts produced by these bacteria, whether from equol producers or nonproducers [17,88]. Intersubject differences in responses to isoflavones may reflect subject-specific differences in gut microfloral species [89]. Chang and Nair [58,59] showed that incubation of daidzein and genistein with fecal bacteria under anaerobic conditions resulted in the conversion of genistein to dihydrogenistein and daidzein to dihydrodaidzein and equol, with equol being the major metabolite detected in the cultures.

Hur and Rafii [22] found that *Eubacterium limosum*, an anaerobic intestinal bacterium capable of producing O-demethylation of several methylated compounds, metabolizes three naturally occurring isoflavones, biochanin A, formononetin and glycitein. The demethylated products, genistein, daidzein and 6,7,4’-trihydroxyisoflavone, respectively, were detected in cultures after incubation with the bacterial cells but not with spent culture medium, indicating that the demethylase activities are cell-associated.

Hur *et al.* [48], screening the fecal bacteria from a healthy individual, found an *E. coli* strain, HGH21, and a Gram-positive bacterial strain, HGH6, that produced daidzein and genistein from daidzin and genistin. Both of these bacteria have glucosidase activity. Strain HGH6 also reduces daidzein and genistein to dihydrodaidzein and dihydrogenistein, respectively, under anaerobic conditions. The reduction of the double bond between C2 and C3 in daidzein and genistein is isoflavone-specific; strain HGH6 does not reduce similar bonds in the flavonoids apigenin and chrysin [48]. Although dihydrodaidzein is an intermediate in the conversion of dihydrodaidzein to equol, no further metabolites were produced in cultures incubated with daidzein or synthetic dihydrodaidzein and dihydrogenistein, indicating that different enzymes and bacteria were required for this process [48].

Hur *et al.* [90] also isolated an anaerobic Gram-positive bacterium, designated as HGH136, by screening human fecal bacteria for daidzein-metabolizing strains. 16S rRNA analysis identified strain HGH136 to be closely related to *Clostridium* species [48]. This bacterium cleaves the C-ring of daidzein to produce *O*-DMA under anaerobic conditions [90]. *O*-DMA is also produced by the incubation of strain HGH136 with synthetic dihydrodaidzein [90]. No other metabolites were found as the result of incubation of strain HGH136 with either daidzein or dihydrodaidzein [90].

Later, another strain of *Clostridium*-like bacteria, designated TM-40, was found by Tamura *et al.* [91] to metabolize daidzin. The strain, which was isolated from the fecal material of a healthy young boy on a Japanese diet, is capable of converting both daidzin and daidzein to dihydrodaidzein. The 16S rRNA sequence of this strain is somewhat similar to *Clostridium* species but has 93% similarity to that of *Coprobacillus catenaformis* AB030218 [91]. Strain TM-40 enhanced the production of dihydrodaidzein in *in vitro* fecal cultures from equol producers and nonproducers, and enhanced the amount of equol produced in the cultures of producers [92].

## 7. Genera of Equol-Producing Bacteria

### 7.1. Eggerthella, Adlercreutzia, and Asaccharobacter

Among the intestinal colonic bacteria that are associated with production of equol are several strains of bacteria with sequence similarity to *Eggerthella* species. Maruo *et al.* [93] isolated nine strains of bacteria, with 16S rRNA genes having 93% similarity to that of *Eggerthella sinensis* that are capable of metabolizing isoflavones by producing equol from daidzein. There were differences in biochemical reactions and cell wall peptidoglycan types between these strains and *Eggerthella*, so they were classified in a new genus and species as *Adlercreutzia equolifaciens* [93]. The type strain, *A. equolifaciens* DSM 19450, has been sequenced [93].

Wang *et al.* [94] isolated a strain, Julong 732, from human colonic bacteria, with 92.8% similarity to *Eggerthella hongkongensis* HKU10 and capable of converting dihydrodaidzein, but not daidzein, to equol. Through further experiments, this strain was shown to produce equol from synthetic (3*S*,4*R*)-tetrahydrodaidzein by a tetrahydrodaidzein reductase, which is sensitive to oxygen and requires NADH for activity [95,96]. However, strain Julong 732 did not produce equol from dihydroequol, indicating that dihydroequol is not an intermediate in the production of equol from daidzein [95,96]. 

Another strain of fecal bacteria, *Eggerthella* sp. strain YY7918, isolated from a human, converts daidzein and dihydrodaidzein to *S*-equol [97]. The genome of this strain has been sequenced [98]. 

An additional strain of bacteria, designated do03T, with similarity to *Eggerthella* and capable of producing equol from daidzein through production of dihydrodaidzein, has been isolated from rat intestine [99]. Butyric acid and arginine enhance the capability of strain do03T to produce equol in a more than four-fold quantity [99]. Due to distinctive biochemical characteristics, it was described as *Asaccharobacter celatus* [99]. 

Recently, some equol-producing bacteria isolated from stinky tofu have been shown by 16S rRNA analysis to have similarities to *Eggerthella sinensis* HKU14 and *Eggerthella lenta* JCM9979 [100,101].

### 7.2. Slackia

Some strains of the genus *Slackia* also metabolize isoflavones [102,103]. A Gram-positive, non-sporeforming bacterial strain, designated NATTS, was isolated from the fecal bacteria of an equol-producing individual. It converted daidzein to equol in cultures containing sorbose as carbon source [102]. Using the 16S RNA sequence, the strain was determined to belong to *Slackia* [102]. Further study showed that the numbers of *Slackia* sp. similar to strain NATTS in feces of Japanese men were correlated with the concentration of equol in the sera of both prostate cancer patients and controls [104]. The genes converting daidzein to dihydrodaidzein, and dihydrodaidzein to equol, from this bacterium were cloned and characterized [103]. They encoded three contiguous open reading frames (Orf 1–3) [103]. Tsuji *et al.* [103] showed that daidzein was converted to dihydrodaidzein by a protein encoded by *orf3*, belonging to the NADH:flavin oxidoreductase family. Dihydrodaidzein is converted to *cis-* and *trans*-tetrahydrodaidzein (THD) by a protein encoded by *orf2*, belonging to the short-chain dehydrogenase/reductase superfamily. *cis* and *trans*-THD are converted to equol by a protein encoded by *orf1* that has an amino acid sequence similar to that of succinate dehydrogenase [103].

A strain of *Slackia isoflavoniconvertens* detected among human colonic bacteria capable of metabolizing isoflavones converts daidzein to equol via the intermediate dihydrodaidzein [105]; the author showed that it transformed 80 µM of daidzein to equol in 10 h under anaerobic conditions. This bacterium also converts genistein to dihydrogenistein, which is then partially transformed to 5-hydroxyequol [105]. Schröder *et al.* [106], using daidzein-induced cultures of *S. isoflavoniconvertens*, identified the proteins involved in daidzein metabolism. They concluded that daidzein reductase converted daidzein and genistein to dihydrodaidzein and dpihydrogenistein; and recombinant dihydrodaidzein reductase and tetrahydrodaidzein reductase reduce dihydrodaidzein to equol [106].

Jin *et al.* [107] isolated a strain of bacteria, designated as DZE, capable of converting daidzein to equol and found that it had 89% and 88% similarity to *Slackia faecicanis* CCUG 48399 and *Slackia exigua* AF101240, respectively, and 87% similarity to *Slackia heliotrinireducens*. They reported that strain DZE converted 150 µM of daidzein to equol in 24 h with a 85% yield and transformed genistein to 5-hydroxyequol [108]. The strain has been named *Slackia equolifaciens* [108].

### 7.3. Lactococcus

Uchiyama *et al.* [109] isolated an equol-producing strain 20–92 from the human intestinal tract and identified it as *Lactococcus garvieae* by 16S rRNA analysis. Genetic analysis has shown that this bacterium has an open reading frame, *Orf US2*, which encodes a dihydrodaidzein reductase that converts daidzein to dihydrodaidzein. This enzyme requires NAD(P)H for activity, is similar to the 3-oxoacyl-acyl-carrier-protein reductases of several bacteria, and belongs to the short-chain dehydrogenase/reductase family [110,111,112]. *L. garvieae* strain 20–92 also has a second open reading frame, *orf-US3*, which encodes tetrahydrodaidzein reductase (THDR) and converts dihydrodaidzein to tetrahydrodaidzein. THDR is similar to several putative fumarate reductase/succinate dehydrogenase flavoprotein domain proteins [110,111,112]. This strain also carries a novel dihydrodaidzein racemase capable of converting both the (*R*)- and (*S*)-enantiomers of dihydrodaidzein to produce the racemate [110,111,112]. Shimada *et al.* [112] conclude that the equol biosynthesis pathway in *L. garvieae* strain 20–92 includes the following steps: L-Daidzein reductase converts daidzein to (*R*)-dihydrodaidzein. A novel dihydrodaidzein racemase rapidly racemizes the (*R*)-dihydrodaidzein and produces (*S*)-dihydrodaidzein. The resulting (*S*)-dihydrodaidzein is then preferentially converted to *trans*-tetrahydrodaidzein by dihydrodaidzein reductase. In the final reaction, L-tetrahydrodaidzein reductase converts *trans*-tetrahydrodaidzein to (*S*)-equol. *L. garvieae* also has been isolated from Italian cheese and appears to be an efficient equol producer [113]. *L. garvieae* has been investigated for use in producing equol-containing compounds that have been tested in experimental animals and humans [34,114].

### 7.4. Other Isoflavone-Metabolizing Genera

Raimondi *et al.* [49] screened 22 strains of *Bifidobacterium* representative of human strains and found that 12 of them efficiently converted daidzin to daidzein. However, none of the strains could convert daidzein to equol. *Bifidobacterium animalis*, *B. longum*-a, and *B. pseudolongum* deconjugate malonyl-, acetyl- and β-glucoside conjugates of daidzin, which are found in soy milk, to produce daidzein. These strains are also reported to transform daidzein to equol in soy milk [115]. 

A mixture containing *Lactobacillus mucosae* EPI2, *Enterococcus faecium* EPI1, *Finegoldia magna* EPI3, and *Veillonella* sp. strain EP, produced equol from daidzein, but none of the strains produced equol in pure culture [60].

## 8. Factors Affecting Production of Equol

In humans, conversion of daidzin and daidzein to equol and *O*-DMA results from the enzymatic activities of specific bacteria from the intestinal tract [54]. The capacity to produce equol and *O*-DMA remains stable over time and may be under genetic control of the host [116]. Van de Merwe *et al.* [116] postulated that the host genetic characteristics are important in determining the resident fecal flora. Brown *et al.* [10] found that production of equol is developmentally regulated and that a higher proportion of infants consuming formula produced equol than of breast-fed infants [10]. In Japanese adults, the positive association of a diet containing fat with higher excretion of equol in urine has been shown [89]. Also, a higher frequency of equol producers has been observed among vegetarians, who are ~4.25 times more likely to be *S*-equol producers than nonvegetarians [66].

Fruit consumption also may contribute to the enrichment of bacterial populations metabolizing daidzein. In a limited study, we showed that fecal bacterial samples from five individuals consuming soy diets varied in the metabolism of daidzein. After consumption of fruits for several months, samples from one in five subjects produced equol and *O*-DMA, and samples from two subjects produced either equol and dihydrodaidzein or equol and *O*-DMA [8]. This observation merits further investigation. 

Use of antimicrobial agents also has been shown to affect *in vivo* and *in vitro* metabolism of daidzein and production of equol [65,66]. Sutherland *et al.* [117] showed that antimicrobial agents from different classes had different effects on the *in vitro* metabolism of daidzein by fecal bacteria from monkeys. Exposure of fecal bacteria to ceftriaxone did not affect metabolism of daidzein by the cultures. Bacteria metabolizing daidzein were eliminated after exposure to tetracycline. Treatment of cultures with ciprofloxacin enriched for the bacteria metabolizing daidzein. Cultures of fecal bacteria from monkeys treated with 4 µg/mL of ciprofloxacin metabolized more daidzein than untreated cultures. Brown *et al.* [10] also have shown that the use of antimicrobial agents affects the production of equol in human subjects. An antimicrobial susceptibility assay indicated that *Eggerthella* sp. strain YY7918, isolated from a human, that converts daidzein and dihydrodaidzein to *S*-equol, was susceptible to aminoglycoside, tetracycline, and new quinolone antibiotics [97]. Other factors affecting daidzein metabolism are short chain fatty acids and arginine. Both butyric acid and arginine enhanced the capability of *Asaccharobacter celatus* to metabolize daidzein by more than four-fold [99]. Age and unknown factors also may affect the metabolism of isoflavones. Recently, Franke *et al.* have shown inconsistencies in the production of equol over time in women, regardless of menopausal status [118,119].

## 9. Metabolic Response to Consumption of Dietary Isoflavonoids

Using nuclear magnetic resonance (NMR)-based metabolomics [120,121], the metabolites in the plasma and urine of subjects consuming soy diets containing isoflavones were analyzed. Consumption of soy resulted in altered levels of trimethylamine *N*-oxide, trimethylamine, choline, creatine, glutamine, and others. The levels of very low density lipoprotein, low density lipoprotein, and ketone bodies (acetate, acetoacetate, and hydroxybutyrate) relative to sugar concentration increased. Other chemicals related to lipid metabolism and biosynthesis also increased, indicating a soy consumption effect on the lipoprotein profile [120,121]. 

Decreases in plasma sugar, increases in lactate, and variations in the plasma levels of glucogenic amino acids (isoleucine and valine) were also observed. The levels of triglycerides were also altered in different subjects consuming soy or daidzein [120,121] indicating changes in carbohydrate and energy metabolism resulting from soy intervention. Solanky *et al.* hypothesized that the increase in lactate and glucogenic amino acids may indicate inhibition of gluconeogenesis [120,121]. They concluded that isoflavones had inhibitory effects on glycolysis, resulting in a shift from carbohydrate to lipid metabolism. The response was subject-specific, and the authors indicated that variation in the colonic microflora and genetic variability influenced the effect of consumption of soy products. Administration of daidzein to subjects with different estrogen receptor genotypes decreased the levels of triglycerides and uric acid in serum to different extents [122]. The contribution of isoflavones from soy in alteration of plasma components, which could influence hormonal activities, is confounded by the fact that soy products contain other components that also may affect the plasma profile [120,121].

## 10. Conclusions

The incidence of a variety of hormone-dependent diseases, including coronary heart disease, atherosclerosis, and menopausal symptoms, is lower in the geographic regions where the population consumes a soy diet. The isoflavones in the soy have similarities to natural estradiols, which has generated interest in consumption of soy-related products for the prevention or alleviation of hormone-related symptoms, including menopause, in women. However, diets containing these compounds have variable results in different subjects, both in the effects and the types of metabolites produced in the individuals. There is a consensus that the differences in the effects for various individuals result from differences in the microfloras of the subjects taking isoflavones. The enzymatic activities of intestinal bacteria are involved in the metabolism, bioavailability, and conversion of isoflavones to compounds with altered estrogenic activities. Substantial research is in progress to find bacteria that produce equol from daidzein, because equol has a higher affinity for the estrogen receptors and a higher biological activity than daidzein. The identification of bacteria converting daidzein to equol should facilitate *in vitro* production of *S*-equol in quantities that could exert biological effects and aid in metabolomic studies using equol.
